# Endometriosis classification/staging and terminology- Are we getting closer to finding a universally accepted language?

**DOI:** 10.52054/FVVO.13.4.040

**Published:** 2021-12-30

**Authors:** Khazali S, Saridogan E

**Affiliations:** HCA The Lister Hospital, Centre for Endometriosis and Minimally Invasive Gynaecology (CEMIG), London, United Kingdom; Ashford & St. Peter’s Hospitals NHS Foundation Trust, Centre for Endometriosis and Minimally Invasive Gynaecology (CEMIG), Chertsey, United Kingdom; Royal Holloway-University of London, Egham, United Kingdom; University College London, Elizabeth Garrett Anderson Institute for Women’s Health, London, United Kingdom; University College London Hospital, Women’s Health Division, London, United Kingdom; NIHR University College London Hospitals Biomedical Research Centre, London, United Kingdom

In this issue, three articles have focused on endometriosis classification and terminology (International Working Group of American Association of Gynecologic Laparoscopists (AAGL), European Society for Gynaecological Endoscopy (ESGE), European Society of Human Reproduction and Embyology (ESHRE) and World Endometriosis Society (WES), 2021a; International Working Group of AAGL, ESGE, ESHRE and WES, 2021b; Hudelist et al., 2021). These articles touch on different aspects of the topic but they all have a common message: a universally accepted endometriosis “language” is urgently needed. A language that will unify us on terminology we use as well as on the way we describe and classify endometriosis in its various phenotypes and severity.

In their consensus paper published in this issue of Facts, Views and Vision, the International Working Group of AAGL, ESGE, ESHRE and WES (2021a) have outlined a list of 49 terms and definitions. We are invited to use ‘deep endometriosis’ instead of ‘deep infiltrating endometriosis’ or ‘DIE’, ‘partial thickness discoid excision’ instead of ‘deep shaving’ or ‘peeling’ and so on. These suggestions were in fact included in previous ESHRE Guideline (Dunselman et al., 2014 - ) and in a joint recommendation by ESGE, ESHRE and WES (Working Group of ESGE, ESHRE and WES, 2021).

There will be disagreement over some of this terminology amongst experts but we believe this is an important step in the right direction. We should leave minor differences of opinions and preferences based on arbitrary historical nomenclature aside to be able to move forward and use the same terminology internationally. The publication of this consensus reached amongst representatives of four large societies that deal with endometriosis is only the start. Changing terminology requires an international effort from all stakeholders for acceptance. If we are to achieve widespread and global change, peer reviewers and editors of scientific journals should insist on the use of this terminology. Similarly, the proposed terminology should be promoted in international conferences and social media. Future editions of relevant textbooks should reflect these changes. We should also reach out to societies not included in this consensus (and there are some large and important societies amongst those) to try and reach a more inclusive agreement globally.

The issue of reaching a universally accepted staging/classification system may be an even more complicated endeavour than the terminology quest.

In the second article in this issue of Facts, Views and Vision, the International Working Group of AAGL, ESGE, ESHRE and WES,;(2021b) have listed and reviewed 22 different endometriosis staging/classification systems that have been proposed to date. Since the manuscript was accepted for publication, another classification system -2021 AAGL Endometriosis classification- has been proposed by Abrao et al. (2021) which is an anatomy-based surgical complexity score. Furthermore, the authors of this editorial are currently running a validation study for yet another system called VNESS (Visual Numeric Endometriosis Scoring system) which focuses on surgical mapping and describing the depth of the disease in 9 different pelvic compartments.

How did we get here? Why have we been unable to come up with a unified system so far? In their discussion, the International Working Group of AAGL, ESGE, ESHRE and WES (2021b) make a very relevant point that “Endometriosis is a challenging disease to classify, as it is known to have different phenotypes and presentations (both with regards to the type of lesions and their location), and various symptoms without a clear link to phenotype or presentation. Moreover, the natural course of the disease is unknown.” This is certainly the main problem. It is not clear whether peritoneal or superficial ovarian endometriosis is a precursor of deep lesions or endometriomas, or whether they develop via completely different mechanisms. The early classification systems were devised when we had little understanding of deep endometriosis - the main challenge for today’s endometriosis surgeon. But these systems enjoyed widespread use mainly because there was nothing better and also because it had the backing of the most influential societies of the time. As we learnt more about this strange disease and different forms it takes, we had to start from scratch, thinking about what we are looking for in the perfect system.

**Figure 1:**
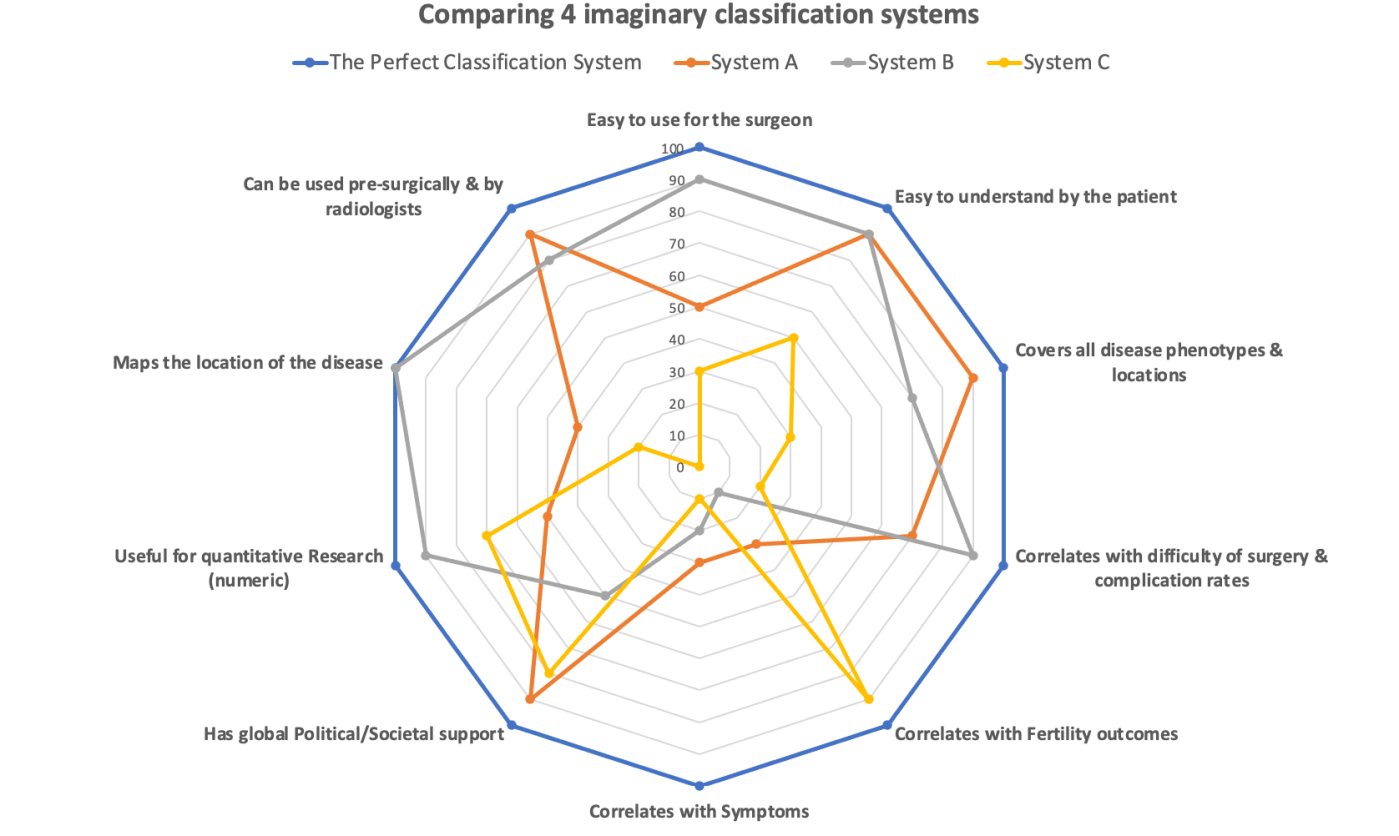
— Components of an ideal endometriosis classification system (blue line) and examples of 3 other imaginary systems indicating how they may perform in individual components.

And what does the perfect solution look like? What do we expect from an ideal system? Figure 1 lists a set of suggestions and plots 4 imaginary systems. The system we choose should be fit for purpose, easy to use and easy to understand. It should certainly be able to describe the severity of endometriosis and the difficulty of surgery. This is important for many reasons; expectation of improvement after medical or surgical treatment, benchmarking for acceptable complication rates, research to compare outcomes and also setting fair remuneration rates -for healthcare systems, hospitals and the surgeon- based on complexity of the procedure performed. This last point is pertinent to public and private systems alike, the importance of which in future progress of our specialty and encouraging high quality establishments and skilled trainees to enter the field should not be underestimated.

We should not forget our most important stakeholder, the patient. Patients expect a commonly accepted classification system to give them an idea of the severity of their disease, in a format that is understandable and familiar.

The “perfect” system that ticks all these boxes is unlikely to be found, due to the nature of the disease but can we get close to it? Can technology help? Electronic patient records have become almost universal and the use of technology to facilitate writing operation records is becoming more and more common. Perhaps with the right technology we can devise a data collection system that converts the “raw data” into various proposed classification systems. We simply describe what we find (on imaging or at surgery) and get a report that includes multiple classification systems. Such technology can also help in finding the best system (or combination of systems). The EQUSUM electronic surgical data collection system seems to perform better than nondigital recording in staging the disease (Metzemaekers et al., 2020) Does the answer lie in finding the right combination of proposed systems? Hudelist et al. (2021) feel this is not practical with the combination of #Enzian, rASRM and EFI and we agree. But we can explore other possibilities.

There is no disagreement amongst experts that we need a universal language. The differences in opinion lie in the answer to the question: “have we found that language yet or should we keep looking?”.

The analogy of “Language”, in the context of classification staging systems, is an appropriate one. There are thousands of languages and dialects spoken worldwide. These languages are part of the culture of people who speak them and are incredibly valuable to them. Languages evolve to reflect the needs of the current times. A language survives only if it is spoken by a large number of people, is taught at schools and are passed down generations. The time has come to move away from the most frequently used rASRM classification as part of the evolution and develop one that will be spoken by the all stakeholders of endometriosis to ensure its survival.
